# Primary intrahepatic cholangiocarcinoma with sarcomatous stroma: case report and review of the literature

**DOI:** 10.1186/s40792-018-0543-z

**Published:** 2018-11-26

**Authors:** Kyohei Yugawa, Tomoharu Yoshizumi, Yohei Mano, Noboru Harada, Shinji Itoh, Toru Ikegami, Yuji Soejima, Nobuhiro Fujita, Kenichi Kohashi, Shinichi Aishima, Yoshinao Oda, Masaki Mori

**Affiliations:** 1 Department of Surgery and Science, Graduate School of Medical Sciences, Kyushu University, Maidashi 3-1-1, Higashi-ku, Fukuoka, 812-8582 Japan; 2 Department of Anatomic Pathology, Graduate School of Medical Sciences, Kyushu University, Maidashi 3-1-1, Higashi-ku, Fukuoka, 812-8582 Japan; 3 Department of Clinical Radiology, Graduate School of Medical Sciences, Kyushu University, Maidashi 3-1-1, Higashi-ku, Fukuoka, 812-8582 Japan; 4 Department of Pathology and Microbiology, Faculty of Medicine, Saga University Hospital, Saga, Japan

**Keywords:** Hepatic carcinosarcoma, Intrahepatic cholangiocarcinoma, Etiology, Radiology and pathology

## Abstract

**Background:**

Hepatic carcinosarcomas, which include both carcinomatous and sarcomatous elements, are uncommon in adults. Although carcinosarcoma in hepatocellular carcinoma is occasionally reported, carcinosarcoma in intrahepatic cholangiocarcinoma (ICC) is an extremely rare ICC variant. Few such cases have been reported in English and no large study of its clinicopathological features exists.

**Case presentation:**

Here, we report a 60-year-old man with an asymptomatic hepatic B infection who developed hepatic carcinosarcoma from an otherwise normal liver. The 6.0-cm tumor was accidentally discovered by PET-CT in a cancer examination. Serum examinations showed no elevation of tumor markers. He underwent left and caudate lobectomy of the liver. The diagnosis of intrahepatic cholangiocarcinoma with sarcomatous stroma was based on thorough pathologic examination and immunohistochemical staining. The tumor exhibited adenocarcinomatous and sarcomatous components; the adenocarcinomatous element was positive for epithelial markers, the sarcomatous element was positive for mesenchymal markers, but negative for epithelial markers. The patient made an uneventful recovery after surgery. At present, 14 months after surgery, he remains well with no evidence of tumor recurrence.

**Conclusions:**

We report an unusual case of hepatic carcinosarcoma (intrahepatic cholangiocarcinoma with sarcomatous stroma) and discuss the etiology and prognosis of this rare disease.

## Introduction

Hepatic carcinosarcoma (HCS) is a rare tumor, which has been defined by the World Health Organization (WHO) as a malignant tumor containing an intimate mixture of carcinomatous (either hepatocellular carcinoma [HCC] or intrahepatic cholangiocarcinoma [ICC]) and sarcomatous elements [1]. The incidence of primary hepatic sarcoma is very low, but sarcomatous change often occurs in several epithelial tumors (including HCC) [2, 3]. Although carcinosarcoma with HCC has occasionally been reported [3–7], few reports of ICC with carcinosarcoma have been reported in English. Because of the scarcity of these reports, preoperative diagnosis of ICC with carcinosarcoma is challenging; little is known about its etiology and prognosis.

We herein present a very rare case of primary intrahepatic cholangiocarcinoma with sarcomatous stroma, confirmed by pathology following resection, and discuss the etiology and prognosis of its radiological imaging and pathology.

## Case presentation

A 60-year-old man was admitted to our hospital with a liver tumor, which was discovered during fluorodeoxyglucose positron emission tomography-computed tomography (PET-CT) as a cancer examination. He had a history of hepatitis B virus infection (positive for hepatitis B virus antigen), but was asymptomatic, showed no positive signs when examined, and had not had any medical interventions.

Analysis of serum tumor markers showed no elevated carbohydrase antigen-19-9 (11.2 U/ml), carbohydrase antigen-125 (18.1 U/ml), or carcinoembryonic antigen (1.0 ng/ml). Other parameter levels were within normal ranges. Gastroscopy and colonoscopy also showed normal findings.

**Fig. 1:**
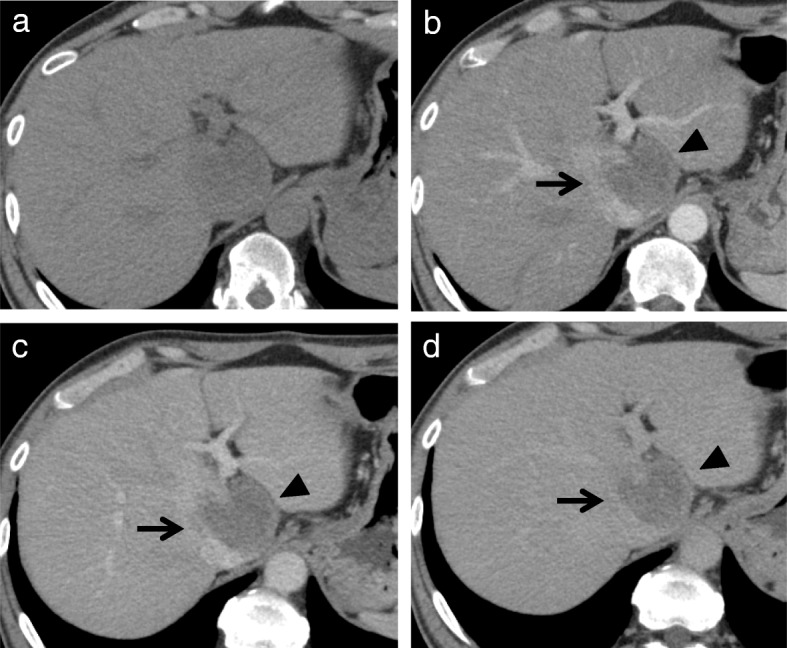
Contrast-enhanced abdominal computed tomography (CT). Plane CT scan shows a well-defined low-density mass (6.0 cm in diameter) in the caudate liver (**a**) Contrast-enhanced CT scan showed right component (arrow) of the tumor enhancement during the arterial phases (**b**) and delayed washout in the latter phases (**c**, **d**), but left component (arrowhead) as hypovascular lesion (**b**–**d**)

**Fig. 2:**
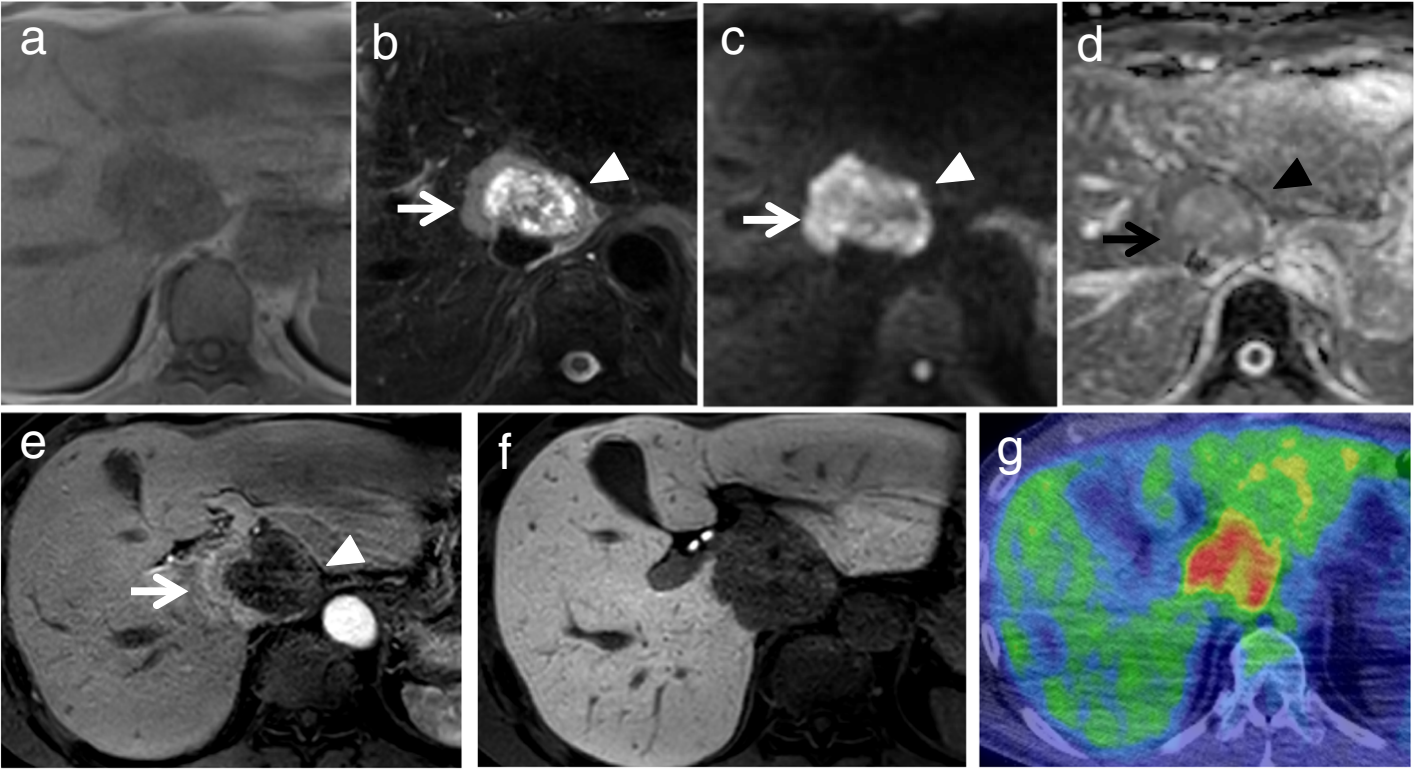
Magnetic response imaging (MRI) and [^18^F]-fluorodeoxyglucose position tomography (FDG-PET). MR images show both components with low intensity on T1-weighted images (**a**) and right component (arrow) of iso-high intensity and left component (arrowhead) of heterogeneously high on T2-weighted images (**b**). DWI showed higher intensity than normal liver parenchyma with a high *b* value of 1000 (**c**). Its ADC value was 1.19 × 10^− 3^ mm^2^/s (arrow on right) and 1.95 × 10^− 3^ mm^2^/s (arrowhead on left) (**d**). EOB-MRI showed right component (arrow) of the tumor as a hyperintense lesion but left component (arrowhead) as a hypointense lesion during the arterial phases (**e**) and hypointense mass in the hepatobiliary phase (**f**). FDG-PET shows accumulation of [^18^F]-FDG at both components (**g**)

Plane computed tomography (CT) scan revealed a well-defined low-density mass, 6.0 cm in diameter, in the caudate liver (Fig. 1a), which showed two different components in the enhanced CT scan. Contrast-enhanced CT scan showed the right tumor enhancement during the arterial phase and delayed washout in the late phase, but showed the left component as a hypovascular lesion (Fig. 1b–d). Magnetic response imaging (MRI) showed both of these components with low intensity on T1-weighted images (Fig. 2a), and right component of iso-high intensity and left component of heterogeneously high on T2-weighted images (Fig. 2b). It also showed higher intensity than with normal liver parenchyma on diffusion-weighted imaging (DWI), with a high *b* value of 1000 (Fig. 2c). Apparent diffusion coefficient (ADC) mean values of these two separated components were 1.19 × 10^− 3^ mm^2^/s (right component) and 1.95 × 10^− 3^ mm^2^/s (left component). It was described as a high-intensity mass on the ADC map (Fig. 2d). Gadolinium-ethoxybenzyl-diethylene-triaminepentaacetic-acid (Gd-EOB-DTPA)-MRI showed the right tumor as a hyperintense in the arterial phase (Fig. 2e) and the whole tumor as a hypointense mass in the hepatobiliary phase (Fig. 2f). [^18^F]-fluorodeoxyglucose positron tomography (FDG-PET) showed accumulation of [^18^F]-FDG at both components (Fig. 2g).

**Fig. 3:**
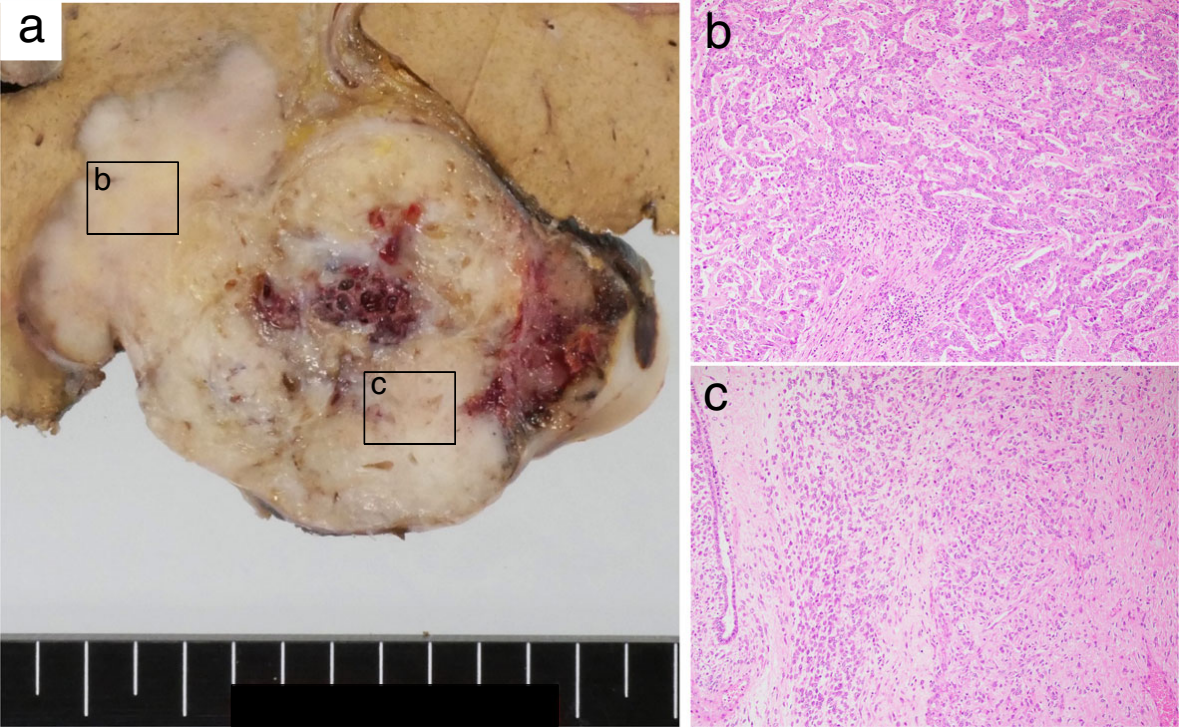
Macroscopic and microscopic findings of sarcomatous ICC. **a** Cut surface shows (right) a well-demarcated, yellowish, nodular lobulated solid component and (left) an elastic soft, cystic component. Micropathologically, **b** the right component was a moderately-to-poorly differentiated adenocarcinoma, with a trabecular and irregular tubular pattern, infiltrated into liver parenchyma (hematoxylin and eosin [HE] staining × 100). **c** The left component was sarcomatous, mainly composed of oval- to spindle-shaped cells with focal dilated gland ductal structure (H&E × 100)

The preoperative diagnosis, based on the imaging studies, was an atypical ICC. After the patient underwent left and caudate lobectomy of the liver, macropathology of the resected specimen showed that the tumor measured 7.5 cm in the largest dimension. The cut surface showed two different components, with a well-demarcated, yellowish, and nodular lobulated solid formation in the right, and an elastic soft and cystic formation on the left (Fig. 3a).

**Fig. 4:**
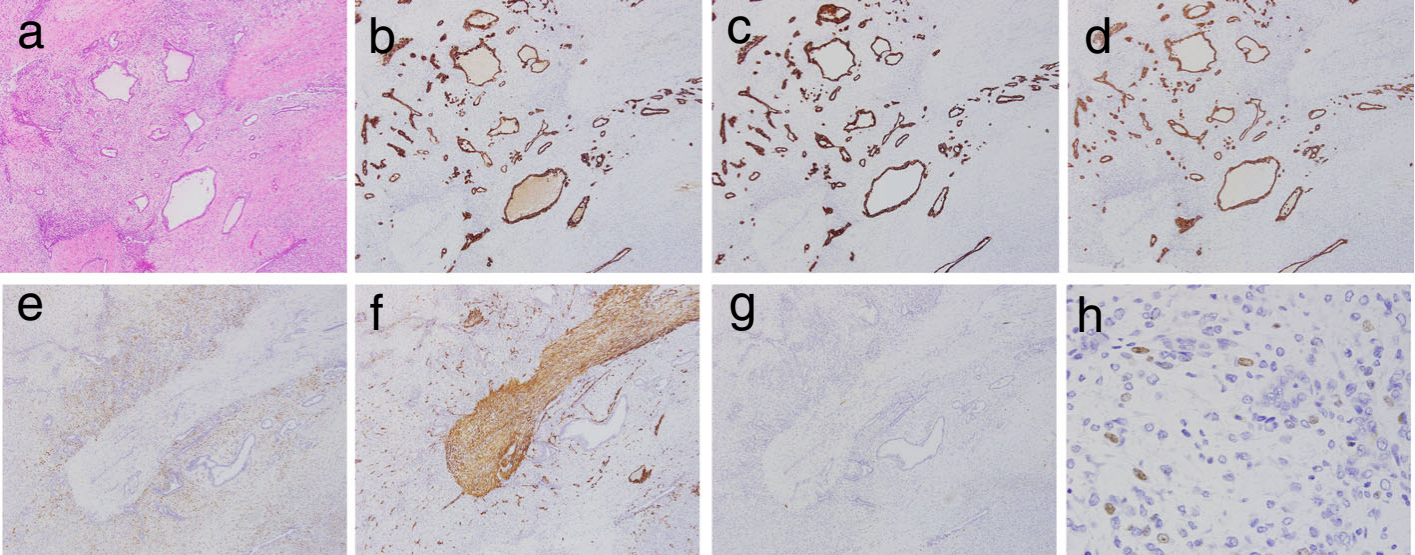
Immunohistochemical staining. Microscopic findings for carcinomatous and sarcomatoid mixed area. **a** H&E staining revealed that adenocarcinoma cells were positive for **b** CK7, **c** CK19, and **d** EMA; and sarcomatous cells were positive for **e** S-100, **f** SMA, and **g** CD10 (**a**–**g**: × 100), but negative for CK7, CK19 and EMA. **h** Ki67 index was 22% in the sarcomatous elements (× 400)

Micropathologically, the right tumor component (indicated as a hypervascular lesion on enhanced CT) showed an adenocarcinomatous element, composed of moderately to poorly differentiated adenocarcinoma, arranged in trabecular and irregular tubular patterns, infiltrated into the liver parenchyma (Fig. 3b). The left component (which appeared with heterogeneous high intensity on T2WI) was a sarcomatous element, mainly composed of oval- to spindle-shaped cells with a focal dilated gland ductal structure (Figs. 3c and 4a). These two components were mostly separate but with a small intermingled area with well-differentiated adenocarcinomatous and sarcomatous elements. There was no evidence of transitional feature between adenocarcinomatous and sarcomatous elements. The surrounding parenchyma showed no cirrhotic change.

In immunohistochemical (IHC) tests, the adenocarcinoma cells were positive for cytokeratin-7 (CK7), cytokeratin-19 (CK19), CD56, and epithelial membrane antigen (EMA), but negative for hepatocellular carcinoma markers such as Glypican-3 (date not shown). There were no histologic elements suggesting HCC. The sarcomatous cells were positive for S-100, α-smooth muscle actin (SMA), and CD10, but negative for CK7, CK19, CD56, and EMA (Fig. 4b–g). The Ki67 index was 22% in the sarcomatous elements (Fig. 4h). These findings led to a pathological diagnosis of carcinosarcoma (ICC with sarcomatous stroma). The patient recovered uneventfully from the surgery, and at present, 14 months later, he remains well with no evidence of tumor recurrence.

## Conclusions

Primary HCS is very rare worldwide, comprising only 1.8% to 9.4% of surgical or autopsy HCC cases [3, 8]. Few cases have been reported in the English language journals and most have been of HCS in HCCs. However, cases of primary HCS in ICCs are even more uncommon and ICC with carcinosarcoma has a much worse prognosis than simple ICC [9].

In 1989, Craig et al. [10] first reported liver carcinosarcomas as hepatic tumors with both an HCC and a non-spindle cell sarcoma and excluded non-hepatocytic epithelial elements. According to the WHO definition, HCS is “a malignant tumor containing an intimate mixture of carcinomatous (either hepatocellular or cholangiocarcinoma) and sarcomatous elements.” Both the WHO and Craig et al. distinguished HCS from collision tumors, and from carcinomas with foci of spindle-shaped epithelial cells, and included tumors designated as “hepatoblastoma, malignant mixed tumor, spindle cell carcinoma, or sarcomatoid carcinoma” [1]. Still, how to distinguish carcinosarcoma from sarcomatoid carcinoma is controversial. Rosai [11] suggested that such mixed tumors should be diagnosed as spindle cell carcinoma or sarcomatoid carcinoma when the sarcomatous component is predominantly composed of spindle cells, but the epithelial cells are still morphologically and immunohistochemically identifiable. Wang et al. [12] suggested the absence of significant differences in survival rates and morphologies of sarcomatous components between sarcomatoid carcinoma and carcinosarcoma, which implies that distinguishing between primary sarcomatoid carcinoma and carcinosarcoma of the liver is clinically unnecessary [12]. Based on our pathological and IHC studies, and according to the definitions of WHO and Rosai, we diagnosed this tumor as “hepatic carcinosarcoma.”

We searched PubMed to identify the published case reports of ICC with sarcomatous change in the English literature and used the terms “liver,” “sarcomatous,” “sarcomatoid,” “carcinosarcoma,” and “cholangiocarcinoma.” We reviewed the identified 27 patients, including our patient, the characteristics of which we here summarize (Table 1) [13–29]. In radiological images, low-density mass with enhancement by contrast medium on CT, hypointensity on T1WI, and hyperintensity on T2WI are reported to be key sarcomatous ICC features [30]. As shown in Table 1, the identified radiological characteristics are similar to those of sarcomatous ICC. As in the previous reports, the radiological images of the distinguished sarcomatous component in the present case might be identical with the dominant sarcomatoid ICC. However, the adenocarcinoma component might differ from the ordinary ICC; hypervascular ICC is considered to have less malignant potential than other ICCs [31]. Nevertheless, the ADC mean value of two different components was 1.19 × 10^− 3^ mm^2^/s (ICC component) and 1.95 × 10^− 3^ mm^2^/s (sarcomatous component), respectively. Lower mean ADC value is associated with more aggressive histopathology and poorly differentiated ICCs [32]. The ADC value indicates that the ICC component has more malignant potential than the sarcomatous component.

**Table 1:** Summarized data on published reports concerning ICC with sarcomatous change

Author	Year	Age (years)/ Gender	Tumor location	Tumor size (cm)	Plain CT	Enhancement CT	T1WI	T2WI	Treatment	Carcinomatous component	Sarcomatous component	Distribution	Transitional feature	IHC of carcinomatous component	IHC of sarcomatous component	Preoperative diagnosis
Sasaki et al. [13]	1991	79/M	Left lobe	8	ND	ND	ND	ND	None	Adenosquamous carcinoma	Spindle and pleomorphic cells	ND	ND	Keratin+, EMA+,	Vim+	ND
Haratake et al. [14]	1992	59/M	Left lobe	Fist-sized	ND	ND	ND	ND	None	Poorly adenocarcinoma	Spindle cells	Intermingled	ND	EMA+, CEA+, CK+	Vim+	Liver abscess
Nakajima et al. [15]	1993	84/F	Hepatic hilum	3.5	ND	ND	ND	ND	None	Moderately adenocarcinoma	Spindle and pleomorphic cells	Intermingled	+	Keratin+, EMA+	Keratin+, EMA+, CA19-9+	ND
		43/F	Right lobe	14	ND	ND	ND	ND	Right lobectomy	Moderately adenocarcinoma	Spindle cells	Intermingled	+	Keratin+, EMA+	Keratin+, EMA+, Vim+	ND
		73/F	Left lobe	7	ND	ND	ND	ND	Anti-cancer chemotherapy	Moderately adenocarcinoma	Spindle and pleomorphic cells	Intermingled	+	Keratin+, EMA+	None	ND
		37/M	Left lobe	10	ND	ND	ND	ND	None	Moderately adenocarcinoma	Spindle and pleomorphic cells	Intermingled	+	Keratin+, EMA+	Keratin+, EMA+, Vim+	ND
		64/M	Left lobe	7.5	ND	ND	ND	ND	TAE	Poorly adenocarcinoma	Spindle and pleomorphic cells	Intermingled	+	Keratin+, EMA+	Keratin+, EMA+	ND
		52/M	Right lobe	7.5	ND	ND	ND	ND	TAE	Poorly adenocarcinoma	Spindle and pleomorphic cells	Intermingled	+	Keratin+, EMA+	Keratin+, EMA+, CEA+	ND
		69/M	Left lobe	10	ND	ND	ND	ND	Left lobectomy	Poorly adenocarcinoma	Spindle cells	Intermingled	+	Keratin+, EMA+	None	ND
Imazu et al. [16]	1995	77/M	Segment 2	6	Low density	Ring enhancement	Low intensity	Low intensity	Lateral segmentectomy	Glandular formation	Spindle cells	Intermingled	ND	Keratin+, CEA+, Vim+,	Keratin+, CEA+, Vim+,	ICC
Honda et al. [17]	1996	61/F	Right lobe	Numerous variously sized	ND	ND	ND	ND	None	Moderately to poorly adenocarcinoma	Rhabdoid cells	Separated (intermingled at the border)	+	Keratin+	Keratin+, CEA-, Vim+	ICC with peritonitis carcinomatosa
Matsuo et al. [18]	1999	77/F	Left lobe	7.7	Low density	Ring enhancement	Iso- to low intensity	Heterogeneous high and low intensity	Left lobectomy	Moderately to poorly adenocarcinoma	Spindle cells	Intermingled	+	EMA+, CK+, CEA+	Vim+, Epithelial markers-	Liver abscess
Itamoto et al. [19]	1999	70/M	Segment 5/6	10.7	Low density	Poor	ND	ND	Right lobectomy	Moderately adenocarcinoma	Spindle cells	Intermingled	+	CA19-9+, EMA+ Keratin+, Vim-	Keratin+, EMA+, Vim-H	Recurrent HCC
Shimada et al. [20]	2000	70/M	Segment 5	3.4	ND	ND	ND	ND	Central bisegmentectomy	Poorly adenocarcinoma	Spindle cells	Intermingled	+	EMA+, Keratin+, CEA+, Vim+	EMA+, Keratin+, Vim+	ND
		55/M	Segment 7/8	6.7	ND	ND	ND	ND	Partial hepatectomy	Moderately to poorly adenocarcinoma	Spindle and pleomorphic cells	Intermingled	+	EMA+, Keratin+	EMA+, Keratin+, Vim+	ND
		74/F	Segment 8	4.0	ND	ND	ND	ND	Right lobectomy	Poorly adenocarcinoma	Spindle cells	Intermingled	+	EMA+, Keratin+, CEA+, Vim+	EMA+, Keratin+, CEA+, Vim+	ND
		64/F	Segment 4	8.0	ND	ND	ND	ND	Left trisegmentectomy	Moderately to poorly adenocarcinoma	Spindle cells	Intermingled	+	EMA+, Keratin+, CEA+, CA19-9+,AFP+, Vim+	EMA+, Keratin+, CEA+, CA19-9+, Vim+	ND
Kaibori et al. [21]	2003	69/F	Lateral segment	20	Low density	Ring enhancement	ND	ND	Lateral segmentectomy	Moderately adenocarcinoma	Spindle and pleomorphic cells	Intermingled	+	ND	Vim+, EMA+, CK+	Leiomyosarcoma
Sato et al. [22]	2006	87/M	Left lobe	4.0	ND	ND	ND	ND	None	Moderately adenocarcinoma	Round cells	Intermingled	ND	CK7+, CK19+, CAM5.2+, CA19-9+	CK7+, CK19+, CAM5.2+, Vim+	ICC
Tsou et al. [23]	2008	69/F	Left lobe	2.5	Low density	Ring enhancement	ND	ND	Segmentectomy	Well to moderately adenocarcinoma	Spindle and pleomorphic cells	Intermingled	ND	ND	CK7+, Vim+	ND
Malhotra et al. [24]	2010	60/F	Segment 5	20	ND	Heterogeneous mass	ND	ND	Lateral segmentectomy	Moderately adenocarcinoma	Pleomorphic spindle cells	Intermingled	ND	CAM5.2, EMA+, AE1/AE3+, CK7+, CK19+, CEA+	Vim+, Epithelial markers-	ND
Inoue et al. [25]	2012	61/M	Left lateral segment	20	Heterogeneous mass	Ring enhancement	ND	ND	Lateral segmentectomy	Moderately adenocarcinoma	ND	ND	+	CK7+, CK19+	Vim+, Keratin-1+	GIST
Nakajima et al. [26]	2012	77/F	Right lobe	14	Low density	Heterogeneous enhancement	Low intensity	Iso- to high intensity	Right hepatic trisegmentectomy and caudate lobectomy	Moderately adenocarcinoma	Spindle cells and chondrosarcomatous change	Intermingled		AE1+	Vim+, Keratin-	CCC or cystadenocarcinoma
Watanabe et al. [27]	2014	62/M	Hepatic hilum	5.0	Low density	Ring enhancement	ND	ND	Extended right hemihepatectomy	Moderately to poorly adenocarcinoma	Spindle and pleomorphic cells	Intermingled	ND	CK+	CK+, Vim+	ND
Kim et al. [28]	2015	67/F	Left lateral segment	4.5	Low density	Heterogeneous enhancement	ND	ND	Left lobectomy	Well to moderately adenocarcinoma	Pleomorphic and spindle cells with osteoclast like giant cell	Intermingled	ND	CK19+	Vim+	ICC
Boonsinsukh et al. [29]	2018	45/M	Right lobe	7.0	Low density	Mild delayed enhancement	ND	ND	Right hepatectomy	Moderately adenocarcinoma	Spindle cells	Intermingled	ND	Vim+, AE1/AE3+, CAM5.2+, CK7+ , CK19+	ND	ICC
Our case	2018	60/M	Caudate lobe	7.5	Low density	Heterogeneous enhancement	Low intensity	Iso- to high intensity	Left lobectomy and caudate lobectomy	Moderately to poorly adenocarcinoma	Spindle cells	Separated (intermingled at the border)		CK7+, CK19+, EMA+	S-100+, aSMA+, CD 10+	ICC

*CK* cytokeratin, *CA19-9* carbohydrate antigen 19-9, *CT* computed tomography, *EMA* epithelial membrane antigen, *ICC* intrahepatic cholangiocarcinoma, *HCC* hepatocellular carcinoma, *IHC* immunohistochemical, *SMA* smooth muscle actin, *Vim* vimentin, *ND* not described

In previous reports, pathologists have proposed two pathogeneses for HCS. One theory, supported by IHC, holds that HCSs develop from multipotent progenitor or stem cells of the liver. This theory indicates dual differentiation by an immature malignant cell and shows that combination tumors may originate from single totipotent stem cell, which differentiates in separate epithelial and mesenchymal directions [33]. The alternative theory, which is based on observation of transitional zones, posits that conventional tumor cells transform or dedifferentiate into sarcomatous components from hepatocellular or cholangiocellular carcinoma. Some reports support the idea that malignant cells might change into multipotent immature cells [34, 35].

In our case, based on IHC findings for our carcinosarcoma specimen, we support the theory that the carcinosarcoma developed from hepatic progenitor cells or stem cells, which differentiates separately into both epithelial and mesenchymal elements. These two different elements were largely separated, although a focal area was intermingled, with small amounts of adenocarcinomatous elements and sarcomatous elements, with no evidence of transitional zones. As shown in Table 1, distribution of these two elements was intermingled and transitional area was observed in the most cases; however, our histological results were different patterns from previous reported cases. Moreover, only the adenocarcinoma cells were invading the hepatic parenchyma, whereas the sarcomatous element proliferated in the caudate without invading, except for the intra-inferior vena cava. In these morphological features, adenocarcinomatous and sarcomatous elements can have different properties. Interestingly, our IHC results revealed that the adenocarcinoma elements were positive for epithelial markers (CK7, CK19, CD56, and EMA) but negative for mesenchymal markers (S-100, alpha-SMA, and CD10), whereas the sarcomatous elements were positive for mesenchymal markers, but negative for epithelial markers. To our knowledge, no previous cases of separated ICC carcinomatous and sarcomatous components shown by radiological and IHC findings have been reported.

In conclusion, we reported an unusual case of hepatic carcinosarcoma (ICC with sarcomatous stroma). The results of the present case report supported the etiological theory that sarcomatous elements developed from progenitor or stem cells, rather than redifferentiated from epithelial elements. More epidemiological and pathological data will be further required to confirm the etiology and prognosis of the rare malignant tumor.

## Abbreviations

ADC: Apparent diffusion coefficient
CK19: Cytokeratin 19
CK7: Cytokeratin 7
CT: Computed tomography
DWI: Diffuse weighted imaging
EMA: Epithelial membrane antigen
EOB-MRI: Gadolinium ethoxybenzyl diethylenetriamine pentaacetic acid-enhanced magnetic response imaging
FDG-PET: [^18^F]-fluorodeoxyglucose position tomography
HCC: Hepatocellular carcinoma
HCS: Hepatic carcinosarcoma
ICC: Intrahepatic cholangiocarcinoma
IHC: Immunohistochemical
MRI: Magnetic response imaging
PET-CT: Fluorodeoxyglucose positron emission tomography- computed tomography
SMA: Smooth muscle actin
WHO: World Health Organization.
